# Recent Strategies for the Immobilization of Therapeutic Enzymes

**DOI:** 10.3390/polym14071409

**Published:** 2022-03-30

**Authors:** Chen-Yuan Zhu, Fei-Long Li, Ye-Wang Zhang, Rahul K. Gupta, Sanjay K. S. Patel, Jung-Kul Lee

**Affiliations:** 1 School of Pharmacy, Jiangsu University, Zhenjiang 212013, China; zhuchenyuann@126.com (C.-Y.Z.); feilongli2018@outlook.com (F.-L.L.); 2 Department of Chemical Engineering, Konkuk University, Seoul 05029, Korea; guptarahul9m@gmail.com

**Keywords:** adsorption, carrier, covalent attachment, entrapment, immobilization, protein engineering, therapeutic enzyme

## Abstract

Therapeutic enzymes play important roles in modern medicine due to their high affinity and specificity. However, it is very expensive to use them in clinical medicine because of their low stability and bioavailability. To improve the stability and effectiveness of therapeutic enzymes, immobilization techniques have been employed to enhance the applications of therapeutic enzymes in the past few years. Reported immobilization techniques include entrapment, adsorption, and covalent attachment. In addition, protein engineering is often used to improve enzyme properties; however, all methods present certain advantages and limitations. For carrier-bound immobilization, the delivery and release of the immobilized enzyme depend on the properties of the carrier and enzyme. In this review, we summarize the advantages and challenges of the current strategies developed to deliver therapeutic enzymes and provide a future perspective on the immobilization technologies used for therapeutic enzyme delivery.

## 1. Introduction

Immobilization processes have been effectively demonstrated to improve the properties of biocatalysts, including whole cells and enzymes, for useful biotechnological applications, such as environmental and medical sectors [1,2,3]. Enzymes have been used as therapeutic agents in the last century. Initially, proteolytic enzymes, such as pepsin were used for the treatment of gastrointestinal disorders, such as lysosomal storage disease. With the development of biotechnology, enzymes have been used to treat a wide range of diseases, including genetic diseases, cancer, infectious diseases, burn debridement, miscellany, and clotting [4].

Owing to their high activity, unique selectivity for target substrates, and catalytic capabilities, enzymes are more advantageous than traditional drugs [5]. Although there are a number of enzymes that could potentially be applied in clinical medicine, only a small amount can be used because of the body’s capacity to develop defensive responses to extraneous proteins, which results in their rapid clearance [6]. As proteins, enzymes are costly and also easily degraded because of their large size and limited distribution within the host [7]. To protect therapeutic enzymes from degradation, scientists have attempted to modify or immobilize enzymes. Immobilization is an important strategy for stabilizing enzymes, especially for the delivery of therapeutic enzymes. In the present review, the various immobilization methods developed for therapeutic enzymes are discussed and summarized.

## 2. Immobilization of Therapeutic Enzymes

There are three main immobilization methods used for enzymes: adsorption, entrapment, and covalent attachment [8,9,10,11]. Rapidly developing strategies, such as molecular techniques, protein engineering, and nanotechnology have also been reported for the immobilization of enzymes with therapeutic applications [3,9,11,12,13,14].

### 2.1. Entrapment

The entrapment of enzymes is an important delivery strategy for therapeutic enzymes. In this method, enzymes can be entrapped in hydrogels, liposomes, red blood cells (RBCs), or deoxyribonucleic acid (DNA) cages and then transported to the target tissue.

#### 2.1.1. Entrapment with Hydrogels

Chitosan and chitin are linear polysaccharides with *N*-acetyl-D-glucosamine and D-glucosamine units at different ratios. Owing to their exceptional ease in forming hydrogels and remarkable affinity for proteins, chitin- and chitosan-based materials have the advantages of good biocompatibility and biodegradability, non-toxicity, and physiological inertness [15,16,17]. Enzymes can be entrapped without requiring chemical activation and cross-linker or bifunctional agents. 

Lipase has been reported to be immobilized inside chitosan hydrogel beads; moreover, the entrapment efficiency was approximately 51%, and the activity of immobilized lipase for freeze-dried beads was 1110 U/mL [18,19]. Chitosan hydrogels have low solubility and are highly pH-dependent, which could lead to undesired enzyme release in the stomach [20]. Therefore, graft copolymerization with acyl, alkyl, monomeric, and polymeric moieties of chitosan is required for applications in enzyme immobilization to allow the entrapped enzyme to be delivered more accurately; for this purpose, magnetite could be introduced for the matrix (Table 1) [21,22,23].

Carboxymethylcellulose (CMC) is also a good carrier for the preparation of hydrogels. Prepared CMC gels could be used as supports to carry superoxide dismutase (SOD) for wound healing, and the resistance of native SOD to hydrogen peroxide inactivation and the effects of applying SOD-CMC hydrogels to open wounds on the backs of rats were examined [24]. The results showed that SOD entrapped in the hydrogel could be used to shorten the wound healing time (19 and 18 days for SOD and SOD-CMC 50%, respectively) and was also more resistant to peroxide inactivation when compared with the native enzyme. CMC can be used to form network beads with alginate for protein entrapment, and the network beads were found to shrink in gastric conditions and swell in intestinal conditions. Therefore, the controlled delivery of an enzyme to a particular part of the gastrointestinal tract was demonstrated [25].

Hydrogels are three-dimensional matrices composed of a cross-linked polymer network and hydrophilic polymer chains that resemble soft human tissue because of the immobilization of large amounts of water [26]. For this reason, hydrogels are one of the best carriers for therapeutics because they have good biocompatibility and can be used for a number of biomedical applications. Degradable dextran hydrogels have excellent biological compatibility and can be used as a long-term delivery system with enzyme release patterns varying over wide time ranges: from days to months [27,28]. 

For example, L-asparaginase, which is used as a chemotherapeutic agent for the treatment of acute lymphoblastic leukemia, could cause side effects, such as allergic or hypersensitivity reactions. However, its immobilization with dextran hydrogels could improve its delivery and eliminate allergic reactions. In addition, to solve the inactivation of enzymes in organic solvents, dextran microspheres could be used, which could be formed in a poly(ethylene glycol) (PEG) water system [29,30]. 

Selecting the proper PEG molecular weight, concentration, and degree of substitution is essential for the entrapment and delivery of enzymes. Dextran can be incorporated with other biomaterials, such as alginate [31] or chitosan [19], for the entrapment of proteins or enzymes. PEG-b-polylactic acid was used for the encapsulation of β-galactosidase as polymersomes (~145 nm) and showed 72% residual activity. The resulting biocatalyst was found to be effective in the treatment of lysosomal storage disorders of the brain [32]. Alkaline phosphatase immobilized through composite bioceramic and hydrogel assembly exhibited enzyme loading up to 48%; thus, it could be a promising candidate for effective drug delivery, especially for bone reconstruction (Table 1) [33].

Alginate is a binary linear heteropolymer containing 1,4-linked β-D-mannuronic and α-L-guluronic acid residues. Barrias et al. [20] reported that entrapped glucocerebrosidase in alginate microspheres retained full activity and exhibited a broader pH-dependent activity profile. Moreover, the optimum pH was shifted toward the alkaline region by approximately 0.5 units, and the enzyme could also be released in a sustained manner. Glucose oxidase (GOX) has also been entrapped in alginate microspheres either chemically or physically [34], and nano-organized coatings were applied on the microspheres to stabilize the immobilized enzyme in a biological environment. Alginate composites can be prepared by incorporating other matrices, such as nano-hydroxyapatite for sustained drug release [23]. If an appropriate alginate, with an optimal pore size and degradation rate, is chosen for the entrapment of biological macromolecules, the release kinetics can be controlled [35].

In addition to these natural biomaterials, biodegradable organic polymers have been used for drug delivery for approximately 40 years. Poly(lactide-co-glycolide) (PLGA) and poly(caprolactone) are two notable biodegradable organic polymers [36,37,38,39]. Giovagnoli et al. [38,40] reported that SOD and catalase could be entrapped in PLGA microspheres and slowly released (Table 1). The immobilized enzymes significantly enhanced the viability and function of cultured neonatal pancreatic porcine cell clusters, suggesting that this technique could provide new strategies for introducing transplant-suitable pancreatic cell tissue for the treatment of type 1 diabetes mellitus. 

When entrapped inside PLGA microspheres without denaturation, enzymes could be released in a sustained manner for 2 months. Giovagnoli et al. [40] also entrapped SOD and catalase in PLGA microspheres using a water/oil/water double emulsion method. Their results showed that immobilized SOD was better stabilized in PLGA microspheres and that hydrogels were useful for delivering SOD in its native form, making this a promising new depot system.

**Figure 1:**
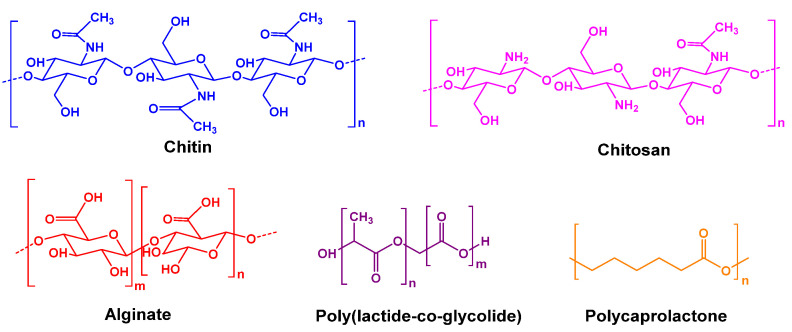
The chemical structures of certain biocompatible hydrogels used for therapeutic enzyme entrapment.

The chemical structures of these naturally degradable hydrogels are presented in Figure 1. All of these hydrogels exhibit common characteristics. They are natural polymers that can be dissolved in water to form hydrogels. Moreover, these hydrogels are typically biocompatible and can be used in the human body without leading to severe rejection reactions. Furthermore, their chemical and physical properties render them as good candidates for the immobilization and delivery of therapeutic enzymes [41].

#### 2.1.2. Liposomal Entrapment

**Figure 2:**
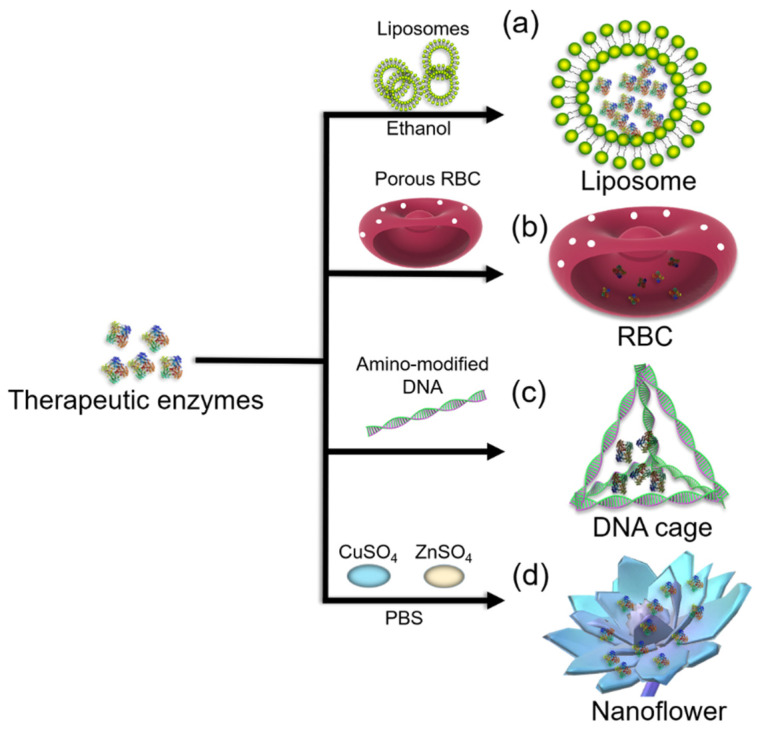
Illustration of entrapment approaches for therapeutic enzyme immobilization using (**a**) liposomes, (**b**) red blood cells (RBCs), (**c**) DNA cages, and (**d**) metal–protein hybrids (nanoflowers). Here, PBS denotes phosphate-buffered saline.

Liposomes are colloidal particles that are formed when phospholipids are dispersed in an aqueous solution [42,43]. Depending on their composition, liposomes can be extremely stable, biocompatible, or biodegradable. Liposome-encapsulated drugs usually have a controlled release pattern, which could be used for the entrapment of enzymes (Figure 2a). The chemical properties, preparation, and applications of liposomes in drug delivery were previously reviewed [44]. In these studies, liposome encapsulation was used to protect β-galactosidase and other enzymes from degradation; it also lowered the risk of immunological reactions and enhanced the possibility of membrane fusion and the delivery of the enzymes into cells [18,45,46]. 

Alternating adsorption of layers of natural polymers, such as alginate, chitosan, and phospholipid vesicles, resulted in enhanced encapsulation efficiency of the protein [46]. The entrapment efficiency depends on the interactions between the enzymes and the phospholipid bilayer. These results indicated that liposomes coated with 10 layers of polyelectrolytes could encapsulate more than 80% of the loaded protein. The enzyme liposome preparation and catalytic efficiency as well as the kinetic properties and stability have also been previously reviewed [46,47]. 

The reverse-phase evaporation technique is widely used for the preparation of liposomes. When this strategy is used, all components that remain after the evaporation of the organic solvent form organogels. Subsequently, nanovesicles were formed via the dispersion of the organogels in pure water under shaking conditions, and liposomes were formed via the addition of the remaining aqueous solution that contained therapeutic enzymes to the organogel under high agitation using a rotary evaporator [48]. The entrapment process had a recondite impact on the enzymes—especially the catalytic efficiency.

#### 2.1.3. RBCs

Compared with extraneous drug carriers, endogenous carriers have various ligands that can recognize targets that are expressed preferentially at the diseased site. Furthermore, they can serve as efficient and safe carriers for targeted drug delivery. Carriers, including RBCs, macrophages, stem cells, platelets, exosomes, albumin, transferrin, and apolipoproteins, were recently reviewed [49]. Among these carriers, RBCs are the most appealing because they are the most abundant cells in human blood, with a lifespan of 100–120 days (Figure 2b). 

Many traditional methods have already been employed to entrap enzymes into RBCs. The most adopted techniques include drug-induced endocytosis, electroporation [50], and dialysis [51]. Only 0.2–0.3% of the added enzymes, such as β-glucosidase and β-galactosidase can be entrapped into RBCs using the hypotonic dilution method. In addition, some higher-molecular-weight substances, such as hemoglobin, ferritin, dextran, and colloidal gold, can enter RBCs using the drug-induced endocytosis method, which presents an entrapment rate of 20%. 

Compared with the dialysis and endocytosis methods, electroporation is based on the decrease in the cell membrane barrier function that occurs upon the exposure of cells to a strong electric field pulse (electropermeabilization), which can significantly increase the permeability of the RBC membrane and lead to higher entrapment efficiency [52]. Using these methods to obtain large pores or perturbations on the cell membrane, several enzymes, including L-asparaginase, acetaldehyde dehydrogenase, and alcohol dehydrogenase, have been successfully loaded into RBCs. He et al. [53] reported that L-asparaginase can be entrapped with cell-penetrating peptides, resulting in a 4% enzyme loading efficiency with about 70% of activity being retained after encapsulation. Compared with the intravenous administration of non-encased β-glucuronidase, RBC-encased enzyme activity was significantly extended [54].

#### 2.1.4. DNA Cage

DNA is an ideal material for the fabrication of rigid structures because its assembly is easy to execute and can be controlled by base-pairing [55]. DNA Td nanocages can be easily prepared and connected by programmable DNA linkers [56]. For the construction of DNA cages, strategies, such as modular self-assembly, the hierarchical assembly of branched DNA tiles, the one-pot approach, and DNA origami-based strategies were previously reviewed [57]. DNA cages can be functionalized with a multitude of chemical tags for target attachment, making them suitable as drug carriers [58]. Erben et al. [59] reported the encapsulation of a single protein molecule into a DNA cage with the protein conjugated to the end of an oligonucleotide through a surface amine. 

This protein–DNA complex was combined with oligonucleotides s2–s4 to form a tetrahedron where the protein molecule was attached to one edge (Figure 2c). In addition, the position of the protein could be controlled by altering the sequence of the edges of the DNA cage [52]. Kim et al. [60] presented a method to encapsulate enzymes in a DNA cage that could transform its conformation depending on the pH, allowing reversible control of the enzyme accessibility to the surrounding environment. 

If DNA cages can be modified with a proper tag, the delivery of encapsulated enzymes to their target is possible. For therapeutic enzymes, the use of DNA cages as immobilization carriers is alluring. Simultaneous loading of cytosine deaminase (as a prodrug enzyme) and doxorubicin (drug) in polymeric nanoparticles performed through tri-block co-polymers containing click nucleic acids can be effectively used for the co-delivery of chemotherapeutic agents for broad therapeutic applications (Table 1) [61].

#### 2.1.5. Metal–Protein Hybrids

Proteins immobilized through the metal–protein hybrid system are known as nanoflowers owing to their flower-like structural morphology [8,9,62,63]. Metal–protein hybrid nanoflowers can be easily synthesized under the harsh conditions required for the modified nanomaterials to immobilize enzymes [64]. Self-assembly of enzyme and copper sulfate at room temperature for an incubation period of 3 days in phosphate buffer showed efficient immobilization of laccase with relative activity up to 650% compared with that of the free enzyme [62]. 

Epinephrine is excreted in the urine of patients with pheochromocytoma, a rare tumor of the adrenal gland. Laccase-based nanoflowers resulted in three-fold faster oxidation of epinephrine than the free enzyme and showed a diagnostic epinephrine detection range of 0.01–1.0 µg/mL. Thus, the detection of epinephrine using laccase-based nanoflowers could reach the diagnostic range of 0.01–1 mg/mL in 24 h [62,63]. Similarly, copper, zinc, calcium, magnesium, cobalt, and manganese based-enzyme immobilization systems have been reported [64,65]. Typically, most nanoflowers are synthesized using copper or calcium ions. 

Although the formation of hybrid nanoflowers using copper ions is facile and does not involve high energy consumption, it is a lengthy process (approximately 3 days). Therefore, it is necessary to shorten the formation time of the hybrid nanoflowers. Zinc ions react with the phosphate radical faster than copper ions. Typically, the formation of biomolecule–inorganic hybrid nanoflowers involves three steps as follows. First, primary copper phosphate crystals are formed. During the growth step, large agglomerates of biomolecules and primary crystals are formed, and flower-like petals appear. 

Finally, branched flower-like structures are formed via anisotropic growth (Figure 2d) [66]. Nanoflower hybrids synthesized using different enzymes exhibited significant improvements in enzyme properties, including efficiency, kinetics, substrate specificity, and stability [9,63]. The effective immobilization of enzymes as nanoflowers has been associated with: (i) large surface areas, (ii) stable structural morphology to reduce significant mass-transfer limitations, and (iii) well-organized confinement of enzyme molecules within the hybrid structure for enhanced co-operative influence [9,63,64]. Metal-based protein hybrid nanoflowers have been applied for efficient drug delivery, as biosensors, and for various medical approaches, including high-resolution images of cells and for monitoring traceable drug delivery systems [62].

Hao et al. [67] reported a mild biomimetic method for calcium hydrogen phosphate (CaHPO_4_) nanoflower synthesis using uricase and horseradish peroxidase (HRP). The hybrid nanoflowers were potentially integrated by a hyaluronic acid dissolvable microneedle system (uricase and HRP-CaHPO_4_@HA MN) to accomplish effective hyperuricemia treatment through transdermal drug delivery. The bio-friendly immobilization of SOD into vaterite calcium carbonate crystals through encapsulation showed a high loading efficiency of 93% [68]. 

The resulting hybrid exhibited controlled release of SOD at physiologically relevant ionic strength, completely retaining its biological activity, which demonstrates that it that can be applied for ophthalmology applications. Similarly, zeolite imidazole framework-8 (ZIF-8) has been used as a promising candidate for biomedical therapies. Bai et al. [69] reported that ZIF-8 was fabricated using the one-pot embedding GOX and HRP strategy (ZIF-8@GOX/HRP) for synergetic cancer therapy. Based on cascade catalysis, ZIF-8@GOX/HRP demonstrated an impressive tumor suppression rate (Table 1).

### 2.2. Adsorption

The adsorption of enzymes onto or into carriers can be accomplished physically or through ionic interactions, allowing for a weak interaction between the enzyme and carrier to be formed. Generally, the immobilization process is mild, and the enzyme properties can be maximally maintained. Thus, many therapeutic enzymes have been immobilized onto a variety of carriers.

#### 2.2.1. Inorganic Carriers

**Figure 3:**
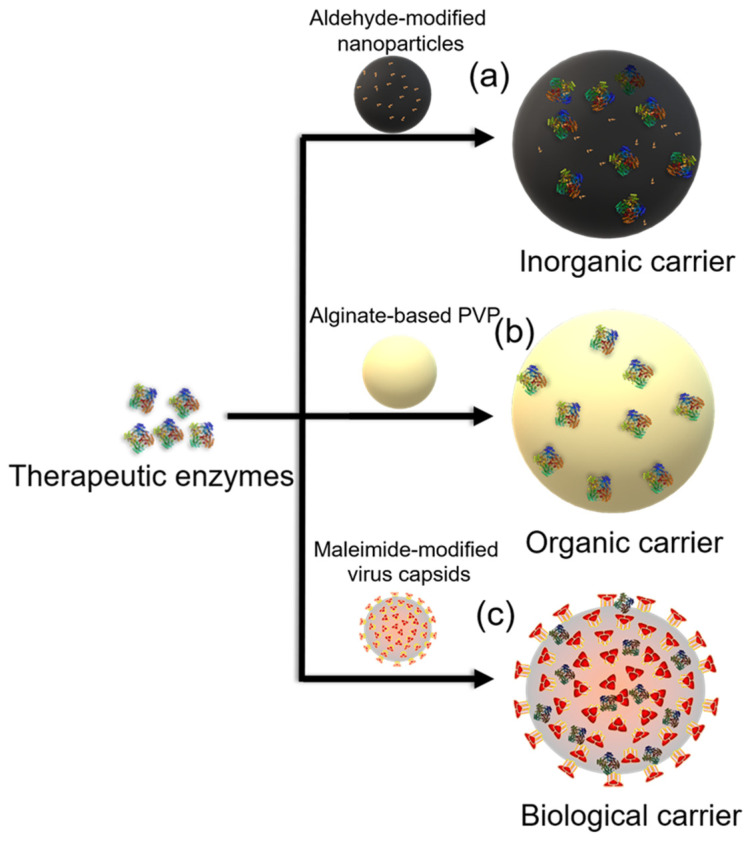
Illustration of adsorption approaches for therapeutic enzyme immobilization on (**a**) silica nanoparticles, (**b**) poly (N-vinyl-2 pyrrolidone) (PVP) microspheres, and (**c**) virus capsids.

Silica nanoparticles are good carriers for the immobilization of therapeutic enzymes because of their good biocompatibility [70] (Figure 3a). The size and shape of nanoparticles will affect the adsorption patterns, protein structure, and function. Many enzymes, such as lysozyme [70], lipase [71,72], nattokinase [73], asparaginase [74], SOD [75,76], β-galactosidase [77], glucocerebrosidase [78], binase [79], hyaluronidase (HAses) [80], GOX, and HRP [69], have been reported to be immobilized onto nanoparticles. Among these enzymes, the activity and stability of glucocerebrosidase are compromised in patients with Gaucher disease (metabolic storage disorder) because these patients do not properly degrade glucosylceramide (GlcCer), which accumulates and can lead to health problems, including bone lesions. 

Enzyme efficiency was significantly increased up to 230% at higher pH values (7–8) after immobilization using self-assembled monolayer nanoparticles (Table 1) [81]. The adsorption of proteins or enzymes onto or into carriers depends on the properties of the enzyme and carrier. The pore size, pH, and temperature are crucial factors for the adsorption of enzymes. Mesoporous silica nanoparticles have been widely explored because of their high stability under physiological conditions, controllable pore size, extensive surface area, and simplicity of synthesis [70]. Metal nanoparticles, such as gold nanoparticles [82] and silver nanoparticles [83] have been fabricated for the immobilization of β-galactosidase and other enzymes. 

Through the surface modification of these nanoparticles, the interaction between enzymes and nanoparticles can be modulated, and their biocompatibility can be improved. Magnetic silica nanoparticles have also received considerable attention because the carried enzyme can be delivered to its target under an external magnetic field. To reduce the toxicity of the magnetic silica nanoparticles, surface modification is required. Patel et al. [84] synthesized a novel type of spherical and porous composite from reduced graphene oxide and magnetic materials to support the immobilization of enzymes. According to the results, this material has the advantages of higher immobilization efficiency and lower acute toxicity. In all the adsorption processes, structural changes may occur, and the enzyme loading will also depend on the surface area [81].

Ordered mesoporous silica nanoparticles have demonstrated effective bioactivity and drug delivery systems owing to their high biocompatibility, broad porosity, and structural morphology [80,85]. Chen et al. [80] reported the stepwise delivery of a biocatalyst and prodrug through multi-responsive nanoplatforms, such as HAase@SiO_2_@prodrug for tumor therapeutics with high efficacy and negligible cytotoxicity of particles toward normal tissue. The synthesized hybrid HAase@SiO_2_@prodrug contained nearly 22% and 48% of HAase and prodrug, respectively. 

Similarly, halloysite nanotubes (HNTs) provide efficient support for the immobilization of SOD and protamine biomacromolecules via self-assembly and act as antioxidant materials [76]. HNTs accommodated 10 and 100 mg of SOD and protamine sulfate polyelectrolyte per gram of particles, respectively. The strong interactions (electrostatic, hydrophobic, and hydrogen bonding) between the HNTs and the biomacromolecules were found to be beneficial to stop enzyme leakage and the HNTs retained two-fold higher residual SOD activity after immobilization [76]. The immobilization method on HNTs with binase, the RNase from *Bacillus pumilus*, displayed a maximum enzyme loading of 85% and a two-fold enhancement in cytotoxicity toward tumor colon cells (Table 1) [79].

#### 2.2.2. Organic Carriers

Organic carriers for the adsorption of therapeutic enzymes in drug delivery systems have also been widely studied. Natural polymers, such as alginate, pectin, chitosan, and carrageenan, have been used for the adsorption of the therapeutic enzymes [5,20,22,86]. Polyhydroxyalkanoate (PHA), an important biodegradable polymer, is synthesized by many Gram-positive and Gram-negative bacteria from at least 75 different genera (Figure 3b) [73,87]. 

When PHA was used as the carrier for the immobilization of nattokinase (which is a fibrinolytic enzyme involved in the hydrolysis of blood thrombi in vivo and the conversion of plasminogen to plasmin) the activity of nattokinase increased by 20% [73]. Moreover, the stability of nattokinase at 4 °C was excellent. Alginate-based biopolymers have also been developed for sustained drug release [23]. In addition to these natural polymers, biodegradable polyesters, such as polylactic acids, poly(caprolactone), and poly(lactic-co-glycolic acid) PLGA, can be synthesized for the adsorption of enzymes [32,40,61]. Compared with natural organic polymers, synthesized polymers exhibit regular and controlled lengths, molecular weights, and specific properties.

Choi et al. [88] reported a biocompatible system of immobilized urease-based nanomotors to treat bladder diseases that can penetrate deeply into the bladder wall and are stable for a long period. The urease-powered nanomotors exhibited 86% residual activity of the free enzyme and showed no cytotoxicity (1 mg/mL) in human bladder cells (RT4). Similarly, alginate was successfully applied for papain immobilization and used as a wound healer because of its accelerating therapeutic features. Papain remained very stable over a long period of 28 days and retained a residual activity of 80% [89]. In contrast, Jamwal et al. [90] reported the synthesis of a novel glucose-responsive system based on GOX and dextran particles that exhibited insulin-controlled release of 90% under artificial intestinal fluid conditions. However, this system can potentially be applied to subcutaneous insulin therapy (Table 1).

#### 2.2.3. Biological Carriers

The natural ability of viruses to adhere to and enter specific cell types could be exploited for the delivery of therapeutic agents to cytosols. Thus, virosomes have also been used as ideal carriers for the transportation of enzymes (Figure 3c) [91]. However, a major limitation of using virosomes as delivery platforms is the potential for a large immune response from the host following exposure to the virosome surface antigen. Extracellular vesicles, virosomes, and liposomes are typical carriers for the delivery of enzymes. As mentioned above, the liposome-entrapped enzyme could have a disease-triggered drug release, and liposomes can remain stable for weeks [92]. 

Bacteria can be used to produce all of the components of extracellular vesicles for the production of complex therapeutics and carriers, which can greatly reduce the cost of production and purification [91]. Recently, a review on liposomes, biopolymers, and virus-like particles for the delivery of enzymes was also presented (Table 1) [92,93]. Compared with synthesized carriers, these biological carriers have more attractive properties, such as high biocompatibility, biodegradability, and adjustable structural characteristics, thus, allowing for a simple and low-cost production strategy.

### 2.3. Covalent Attachment

#### 2.3.1. Conjugation onto Polymers

Polymers are usually used as carriers for the immobilization of therapeutic enzymes, and the immobilized enzyme can be delivered in vivo or used for wound healing. Poly(N-vinylpyrrolidone) (PVP) and poly(vinyl alcohol) (PVA) have been reported for the conjugation of proteins or enzymes, such as SOD [78]. Compared with PVP and PVA, PEG is the standard polymer for the conjugation of enzymes because it results in decreased enzymatic degradation of its payload and reduced immunogenicity. Initially, PEG was produced via the reaction of mono-methoxy-PEG (molecular weight (Mw) 5000) with cyanuric chloride to form PEG dichlorotriazine. 

**Figure 4:**
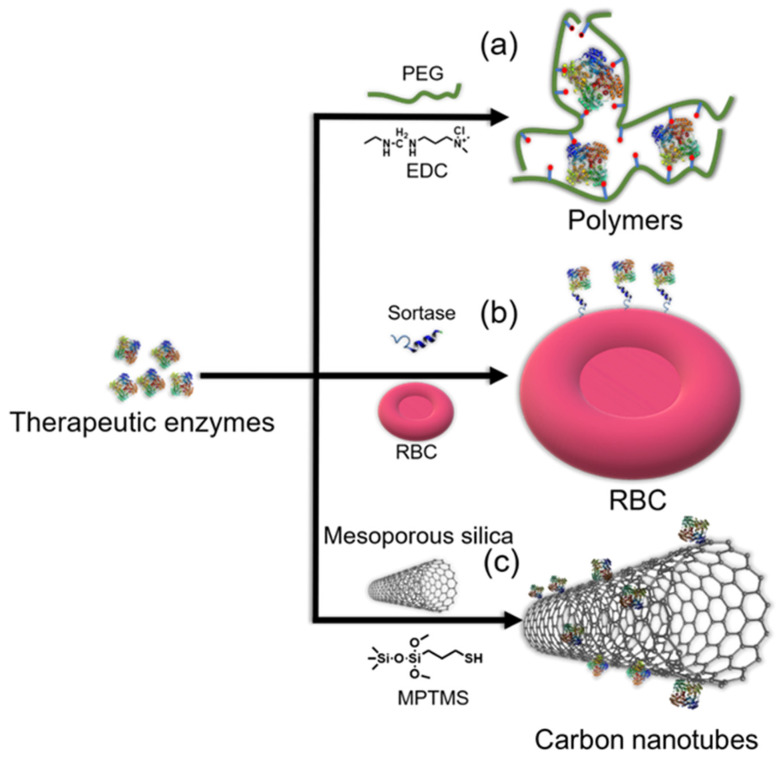
Illustration of covalent approaches for therapeutic enzyme immobilization via conjugation onto (**a**) polyethylene glycol (PEG), (**b**) red blood cells (RBC), and (**c**) mesoporous silica. Here, EDC and MPTMS denote 1-ethyl-3-(3-dimethyl aminopropyl)carbodiimide and mercaptopropyltrimethoxysilane, respectively.

One of the two chlorine atoms of PEG dichlorotriazine would then be displaced by nucleophilic amino acid units, such as lysine, serine, tyrosine, cysteine, and histidine. The remaining chlorine atom was not electrophilic but could react to cause nonspecific crosslinking of the protein; thus, multiple PEG units would become attached to the protein (Figure 4a) [94]. There are two methods for the conjugation of proteins onto polymers: anionic and cationic routes. Furthermore, the prepared samples were also nearly monodispersed. In the past 20 years, several PEGylated enzymes have been approved by the Food and Drug Administration. 

With the development of polymer preparation, the conjugation of enzymes onto polymers will become simpler. In previous studies, PEGylation was used to significantly increase the stability of enzymes, decrease immunogenicity, and prolong circulation time. However, the non-biodegradability of the polymers is a significant limitation of PEGylated enzymes, and this limitation also drove the development of new degradable polymers [52]. 

Magnetic polymer nanoparticles were synthesized by the polymerization of iron oxide (Fe_3_O_4_), 2-hydroxyethyl methacrylate, and glycidyl methacrylate to covalently immobilize L-asparaginase [95]. After immobilization, L-asparaginase retained 74.7% residual activity in artificial serum medium. It covered 50% activity after 10 h at 55 °C and retained 85% activity after eight cycles of reuse, which can be potentially applied for cancer treatment. The covalent immobilization of lysozyme on a polymeric agarose matrix provided beneficial potential as a bacteriolytic activity that can be effectively used against pathogens (Table 1) [96].

#### 2.3.2. Covalent Immobilization onto Nanocarriers

Nanocarriers, especially nanoparticles [97,98] and carbon nanotubes (CNTs) [99], are promising supports for the immobilization of enzymes because they have a large surface area for drug delivery. Ansari and Husain [98] compiled a list of nanomaterials used for the immobilization of enzymes, and the potential for the immobilization of therapeutic enzymes has been widely explored. Inorganic and organic nanocarriers, such as silica and ferric oxide nanoparticles, chitosan, and alginate nanomaterials as well as CNTs with different sizes, have been prepared and modified for the immobilization of enzymes (Figure 4b) [99]. 

For example, Fe_3_O_4_@chitosan nanoparticles have been used as carriers for the immobilization of penicillin G acylase [22]. This is an important enzyme for the pharmaceutical industry; it is used to hydrolyze penicillin G to produce 6-aminopenicillanic acid and 7-amino deacetoxycephalosporanic acid and to synthesize β-lactam antibiotics. The immobilized penicillin G acylase presented higher activity, better reusability, and higher thermal stability compared with the free penicillin G acylase over broad pH and temperature ranges. Many enzymes, such as GOX [100] and HRP [101], have been immobilized on nanoparticles as biosensors with the advantages of high sensitivity, fast response, low cost, small size, and low weight, and have been used in clinical applications. 

The shape and facet configuration influences the enzyme loading and release. Immobilized enzymes on nanoparticles showed a broader working pH and enhanced stability compared with the native enzyme [97]. The hybrid nano-biocatalyst synthesized by biomimetic silica (Si) nanoparticles, magnetic nanoparticles (MNP), polyethyleneimine (PEI), and HRP as BioSi@T_HRP_MNP_PEI exhibited high enzyme immobilization of 78% [102]. Remarkably, the resulting hybrid BioSi@T_HRP_MNP_PEI improved the thermal stability of the enzyme ~280 times, which can potentially be used in directed enzyme prodrug therapy (DEPT) and other related biotechnological applications (Table 1).

#### 2.3.3. Albumin and RBCs as Carriers

Albumin and RBCs are natural carriers and can remain in the blood for between two to three weeks, which is far longer than any polymer [103,104]. As versatile carriers for therapeutic or diagnostic enzymes, enzymes being carried on albumin have been applied for the diagnosis and treatment of cancer, diabetes, and infectious diseases [103]. Enzymes can be engineered to bind, conjugate, or genetically fuse to albumin [104]. 

For example, human butyrylcholinesterase [105] and other molecular drugs, such as hormones, cytokines, growth factors, and peptides, have been reported to be fused to albumin with half-lives far longer than their unfused counterparts. The surface of RBCs can be modified to contain modified proteins on the plasma membrane, which can be labeled in a sortase-catalyzed reaction under native conditions without inflicting damage on the target membrane or cell (Figure 4c) (Table 1) [106]. However, the limitations of using RBCs as carriers include the macrophage-mediated destruction of enzymes-carrying RBCs from long-term circulation, the possibility of enzymes damaging the cells, membrane leakage, and the inability to control the release of enzymes.

**Table 1:** Descriptions of various therapeutic enzymes immobilized through different methods.

Techniques	Support	Enzyme	Descriptions	Reference
Encapsulation	Alginate	Glucocerebrosidase	The immobilized enzyme presented broader activity than the free enzyme at higher pH values. Localized delivery of the enzyme was observed; 40% of the enzyme was loaded within microspheres after 24 h.	[20]
	PEG-b-polylactic acid	β-galactosidase	Polymersomes showed 72% efficiency and restricted release at physiological pH (7.4). Enzymatic-based treatment to the brain in lysosomal storage disorders was demonstrated.	[32]
	Calcium carbonate	Alkaline Phosphatase	Enzyme loading of up to 48% was observed on particles. This approach could be a promising candidate for effective drug delivery, especially for bone reconstruction.	[33]
	Poly(D,L-lactide)	Catalase and superoxide dismutase	Compared with the control enzymes, the entrapped enzymes enhanced the in vitro viability and function of isolated neonatal pancreatic porcine cell clusters.	[38,40]
	Mouse RBCs	Human erythropoietin	Human RBCs presented higher encapsulation yield (22%) than mouse RBCs (14%) and an efficient cell recovery of 70%. The stability of the encapsulated enzyme was highly dependent on the experimental immobilization conditions. Carrier RBCs presented higher hypoosmotic resistance than regular RBCs, and they efficiently released the immobilized enzyme in suspension.	[51]
	PEG-CNA-PLGA	Cytosine deaminase	PEG-CNA-PLGA nanoparticles are efficiently used for the co-immobilization of doxorubicin (a chemotherapy drug) and cytosine deaminase (prodrug enzyme). This system could be used for the codelivery of chemotherapy agents.	[61]
	CaHPO_4_ nanoflower	Uricase and HRP	The synthesized hybrid, uricase and HRP-CaHPO_4_@HA MN, showed maximum encapsulation up to 71%. The resulting hybrid could be used for the treatment of hyperuricemia.	[67]
	Calcium carbonate crystals	Superoxide dismutase	A high loading efficiency of 93% was achieved on particles, and they exhibited an excellent controlled release of the enzyme at physiologically relevant ionic strength without changes in its biological activity. It could potentially be used for SOD delivery in ophthalmology.	[68]
	ZIF-8	GOX and HRP	ZIF-8@Gox/HRP preserved high residual activity up to 2.1 fold. The hybrids showed selective tumor cell growth inhibition.	[69]
	Magnetic hydrogel	L-asparaginase	The immobilization yield of the enzyme was as high as 90%. When the enzyme was immobilized within the polymeric shell, its activity remained unchanged after six months of storage at 4 °C. Moreover, the high biocompatibility of the support can be helpful for the delivery of the enzyme to tumor tissues.	[74]
Adsorption	Polyhydroxyalkanoate	Nattokinase	Enzyme activity increased by 20% after immobilization; moreover, the immobilized enzyme was stable up to 70 °C and did not lose its residual activity after 25 days of storage at 4 °C.	[73]
	HNT-PSP	Superoxide dismutase	HNT-PSP-SOD showed a two-fold higher residual activity without any enzyme leakage. This system can potentially be applied for inflammatory bowel disease therapy.	[76]
	HNTs	Binase	An enzyme loading of 85% was achieved on HNTs, and it exhibited a two-fold enhanced cytotoxicity toward tumor colon cells.	[79]
	Self-assembled monolayers	Glucocerebrosidase	Enzyme efficiency significantly increased up to 230% at higher pH (7–8) values after immobilization.	[81]
	Polydopamine	Urease	The biocompatible properties and long-lasting effectiveness of this system can be used for various bladder disease diagnoses.	[88]
	Alginate	Papain	The enzyme showed stability over 28 days with 80% residual activity. It improved the therapeutic features and thereby increased wound healing.	[89]
	Acryloyl crosslinked dextrandialdehyde	Glucose oxidase	In vitro insulin controlled release of about 70% under artificial intestinal fluid conditions was demonstrated. It can be effectively used for therapeutic applications.	[90]
	Immuno-virosomes	Lysozyme	Approximately 75% of the enzyme cargo was delivered to the cytoplasm, avoiding the endocytic pathway.	[92]
Covalent	Poly(ethylene glycol)	Adenosine deaminase	The PEGylation process dramatically increased the stability of the enzyme, decreased the immunogenicity, and prolonged the circulation time.	[52]
	Fe_3_O_4_@chitosan	Penicillin G acylase	The immobilized enzyme presented higher activity, better reusability, and higher thermal stability than the free enzyme over wide pH and temperature ranges. The conversion yield of 72% was recorded for the synthesis of amoxicillin using the immobilized enzyme at 25 °C.	[22]
	Magnetic poly (2-hydroxyethyl methacrylate and glycidyl methacrylate)	L-asparaginase	Immobilized enzyme retained 50% activity after 10 h under thermophilic conditions (55 °C) and 85% residual activity preserved after 8 cycles of reuses. It could be a promising candidate for cancer treatment.	[95]
	Polymeric agarose matrix	Lysozyme	Almost complete immobilization achieved. It is a promising candidate for the synthesis of complementary material for medical applications in extracorporeal therapy.	[96]
	BioSi@THRP_MNP_PEI	HRP	The immobilization yield was achieved up to 78% and the stability improved ~280 times. It can potentially be used for DEPT and other related biotechnological applications.	[102]
	Mouse RBCs	L-asparaginase	The immobilization of enzymes on RBCs lowered the development of antibody titers by >1000-fold and extended the pharmacodynamic effects of the enzyme drug by approximately ten-fold when administered to mice.	[106]

## 3. Protein Engineering

Protein engineering can be used to modify the primary amino acid sequence of enzymes to improve enzyme structure, stability, and function. By combining protein engineering and immobilization, enzyme properties can be efficiently improved [14,107]. Protein engineering allows site-specific immobilization of enzymes at any desired residue. Smith et al. [108] developed a protein residue-explicit covalent immobilization technique for a stability enhancement system, which uses an amber codon substitution to enable rationally directed non-canonical amino acid incorporation for site-specific immobilization of enzymes. 

This can be used to effectively improve stability during covalent immobilization. Later, the same group used this method to immobilize lysozymes with orientation control, allowing for more than a 50% improvement in activity compared with the randomly immobilized enzymes [109]. Raliski et al. [110] reported the utilization of unnatural amino acid technologies to introduce biorthogonal handles in a site-specific fashion for protein immobilization. When introducing genes into RBCs encoding surface proteins that can be covalently and site-specifically modified on the cell surface, the RBCs can be used as carriers for the immobilization of enzymes, including sortase for drug delivery [106]. 

Compared to traditional immobilization methods, protein engineering allows for orientation-control immobilization [111]. The immobilized enzyme often has high stability and recovery because the immobilization process is totally designable and controllable. The genetic modification of xenobiotic reductases (XenB) from *Pseudomonas putida* facilitated effective immobilization on the gold surface for potential human use in cancer prodrug (CB1954) therapy as a chemotherapy strategy via magnetic nanoparticle-based DEPT [112].

## 4. Conclusions

Compared with industrial enzymes, therapeutic enzymes are more specialized regarding their potential applications in humans. To safely use therapeutic enzymes, immobilization techniques must be reliable, non-toxic, and without side effects. Biocompatibility with the human body is the most important consideration when immobilization is performed. Thus, natural and artificial biopolymers are still the primary choice for the adsorption or entrapment of therapeutic enzymes. 

Due to their high carrying capability as well as easy and controllable preparation methods, nanocarriers will likely become widely used in clinical and diagnostic applications. With the development of computational and molecular techniques, protein engineering will also fulfill important functions in the immobilization of enzymes. The combination of protein engineering and specific immobilization will make the targeted delivery of therapeutic enzymes much easier.

## Data Availability

Data sharing not applicable.
